# Depiction of Delirium in Contemporary Medical Television Dramas: A Descriptive Content Analysis

**DOI:** 10.1111/jgs.70565

**Published:** 2026-06-22

**Authors:** Morgan Guest, Brooke Johnson, Molly Pistono, Jacqueline M. Kruser, Eduard E. Vasilevskis, Blair P. Golden

**Affiliations:** ^1^ Department of Medicine University of Wisconsin‐Madison School of Medicine and Public Health Madison Wisconsin USA; ^2^ Division of Hospital Medicine University of Wisconsin‐Madison School of Medicine and Public Health Madison Wisconsin USA; ^3^ Division of Allergy, Pulmonary, and Critical Care Medicine University of Wisconsin‐Madison School of Medicine and Public Health Madison Wisconsin USA

**Keywords:** acute care, delirium, public health

## 1 Introduction

Delirium is a clinical syndrome characterized by acute, fluctuating disturbances in attention and awareness, often accompanied by changes in level of consciousness [1, 2]. Delirium affects approximately one in four hospitalized adults and is associated with serious adverse health impacts, including accelerated cognitive decline [1, 2]. Prior work suggests the public is unfamiliar with and has important misconceptions about delirium, which could contribute to under‐recognition and mismanagement [3]. These misconceptions may be influenced by wide‐reaching media, including popular television (TV) shows [4, 5]. We aimed to characterize portrayals of delirium in medical TV dramas.

## 2 Methods

We performed a descriptive content analysis of the most recent, complete seasons of 6 medical TV dramas airing between 2022 and 2025 (*Grey's Anatomy, Chicago Med, The Pitt, New Amsterdam, The Good Doctor, The Resident*). After pilot testing a structured abstraction guide and reaching high inter‐rater reliability between two abstractors (kappa > 0.8) for the initial five episodes, data from the remaining episodes were independently abstracted. Attending physicians with delirium expertise arbitrated uncertain assessments. This project was considered non‐human subjects research by the University of Wisconsin‐Madison institutional review board.

For each patient appearing on screen for ≥ 10 s, we assessed depicted level of consciousness using the Richmond Agitation‐Sedation Scale (RASS), which is 64% sensitive and 93% specific for delirium when abnormal [6]. For patients with fluctuating levels of consciousness, we recorded each RASS score as a unique observation. We categorized each patient's depicted age (Pediatric: < 18, Younger adult: 18–64, Older adult: ≥ 65) and noted if they had additional symptoms of delirium (e.g., inattention, acute cognitive changes) [7]. We also recorded instances of the clinical team using the term delirium.

We purposively sampled observations with an abnormal RASS score plus additional evidence of delirium (i.e., inattention) for duplicate review to characterize whether and how common features of delirium were depicted. We excluded comatose patients (RASS −4 or −5) from this qualitative analysis because core features of delirium cannot be assessed. Higher‐level themes were developed and refined within a group of physicians with expertise in delirium.

## 3 Results

Within 84 episodes, there were 376 patients appearing on screen for ≥ 10 s and 695 unique observations with evaluable RASS scores (median: 1, IQR: 1–2 evaluable RASS scores per patient) (Figure 1). The most frequently observed RASS scores were 0 (*n* = 348, 50%; meaning alert and awake) and −5 (*n* = 130, 19%; meaning comatose and unresponsive). Younger adults accounted for the largest proportion of observations.

**FIGURE 1:**
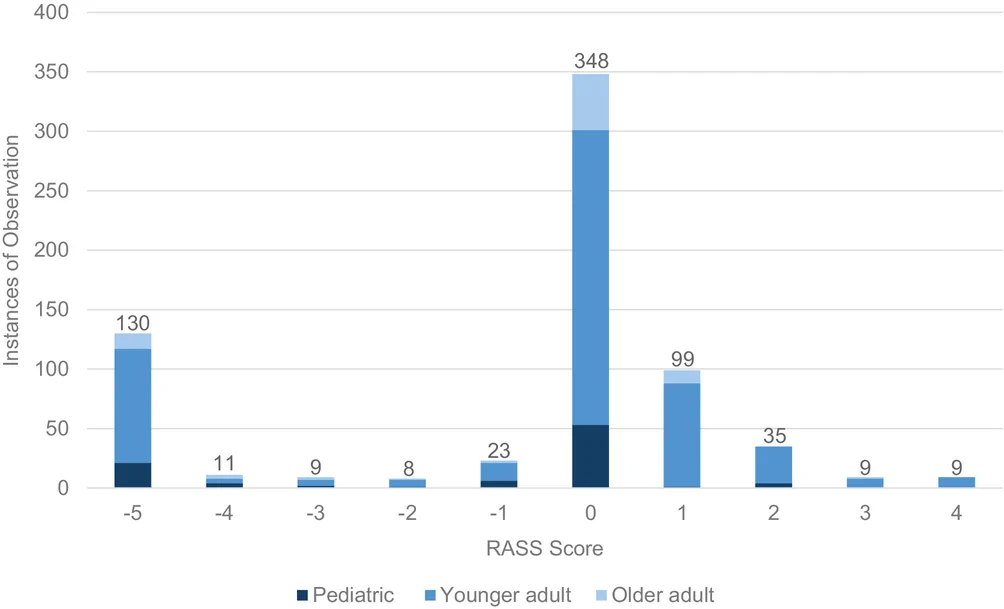
Distribution of Richmond Agitation‐Sedation Scale (RASS) scores across all observations, stratified by age group (*n* = 695).

We identified several themes characterizing whether and how common features of delirium are depicted in TV medical dramas, with potential implications for public understanding (Table 1). Overall, depictions suggestive of delirium were rare and infrequently involved older adults; only one patient with dementia was shown having symptoms consistent with delirium. Across all episodes, the word “delirium” was used once, and the patient was not shown as having core symptoms of delirium. Patients depicted as having potential symptoms were commonly young adults experiencing acute intoxication or individuals with acute neurological conditions (e.g., seizure).

**TABLE 1:** Common features of television depictions suggestive of delirium and potential implications for public understanding.

Features of televised depictions	Representative examples	Potential implications for public understanding
Depictions suggestive of delirium were infrequent and rarely involved patients with pre‐existing cognitive impairment	*Delirium infrequently depicted*: Patient abruptly develops abrupt nonsensical speech, agitation, and fluctuating level of consciousness due to medication‐induced lactic acidosis (The Resident, Season 6, Episode 5)	– Under‐recognition and failure to identify delirium as a medical emergency in high‐risk populations (older adults and adults with dementia).
	*Delirium rarely shown in older adults with cognitive impairment*: Older adult with dementia develops worsening confusion, disorientation, and inattention (The Pitt, Season 1, Episode 2)	
The term “delirium” is rarely used and may not be used to describe clinical syndrome	Patient is described as having “delirium” from a scorpion bite with associated pancreatitis. He requires intubation due to acute respiratory failure but is not shown as being confused or inattentive prior to being intubated (The Resident Season 6, Episode 9)	– Lack of awareness of delirium as a distinct clinical syndrome and not just a synonym for confusion.
Many patients with acutely altered levels of consciousness are intoxicated, young adults	*Alcohol‐induced*: A young adult patient has disinhibited speech and agitation due to alcohol intoxication (Grey's Anatomy Season 20, Episode 5)	– Expectation that symptoms of delirium may be readily treatable and reversible. – Normalization of symptoms in young patients, despite higher prevalence of delirium in older adults.
	*Other substances*: A young adult patient has paranoid delusions and hallucinations after consuming marijuana (New Amsterdam, Season 5, Episode 8)	
Acute neurologic causes are common causes for symptoms	*Seizure*: Patient develops sudden confusion and nonsensical speech preceding seizure (The Resident, Season 6, Episode 5)	– Expectation that symptoms of delirium are due to single precipitant, despite multifactorial etiologies.

## 4 Discussion

Delirium is a serious and distressing medical condition commonly affecting hospitalized patients but is rarely depicted or discussed on medical TV dramas. Patients on medical TV dramas are typically depicted as either fully alert or comatose, reflecting a more simplified representation of alertness among hospitalized patients than what occurs in real‐world practice. Furthermore, TV‐portrayed patients with symptoms of delirium are overwhelmingly young, contrasting sharply with the epidemiology of this condition [1].

Strengths of this study include the use of a validated tool (RASS) to quantify patient depictions across multiple medical dramas. Our study also has limitations. We observed overt depictions of delirium but could not perform gold‐standard delirium assessment, including testing for inattention. Delirium symptoms fluctuate and may not have been captured during on‐screen appearances; however, our goal was to determine if and characterize how delirium is being depicted, not its actual incidence.

In conclusion, medical TV dramas may foster incorrect expectations about delirium, including the notion that it typically has a single, readily reversible precipitant. Depicting altered mental status as primarily occurring in young patients with intoxication or in acute neurologic changes may limit awareness of the more common delirium symptoms affecting older adults. More accurate and age‐concordant portrayals of delirium in popular media may improve public recognition of and understanding about this condition.

## Author Contributions

Data collection: M.G., M.P., B.P.G. Data analysis: All authors. Manuscript drafting: All authors. Final review of the manuscript: All Authors.

## Funding

Dr. Golden's work is supported by the National Institute on Aging under Award Number K23AG081458. The content is solely the responsibility of the authors and does not necessarily represent the official views of the National Institutes of Health.

## Disclosure

The sponsor played no role in the design, methods, subject recruitment, data collection, analysis or preparation of this paper.

## Conflicts of Interest

The authors declare no conflicts of interest.
